# *STAT3^R152W^* Mutation Model Reveals Temporal Changes in Hematopoietic Populations

**DOI:** 10.3390/ijms27031587

**Published:** 2026-02-05

**Authors:** Jakub Jankowski, Jichun Chen, Sung-Gwon Lee, Chengyu Liu, Neal Young, Lothar Hennighausen

**Affiliations:** 1 Section of Genetics and Physiology, Laboratory of Cell and Molecular Biology, National Institute of Diabetes and Digestive and Kidney Diseases, US National Institutes of Health, Bethesda, MD 20892, USAlotharh@niddk.nih.gov (L.H.); 2 Hematology Branch, National Heart, Lung, and Blood Institute, US National Institutes of Health, Bethesda, MD 20892, USA; chenji@nhlbi.nih.gov (J.C.); youngns@nhlbi.nih.gov (N.Y.); 3 Transgenic Core, National Heart, Lung, and Blood Institute, US National Institutes of Health, Bethesda, MD 20892, USA; liuch@nhlbi.nih.gov

**Keywords:** STAT3, germline mutation, missense mutation, immunology

## Abstract

Inconsistent presentation of *STAT3* variants in clinical settings makes them challenging to use in diagnostics and the prevention of unfavorable outcomes. Patients harboring the *STAT3^R152W^* variant display a range of autoimmune disorders, including type 1 diabetes, hemolytic anemia, and thrombocytopenia. Because of a complex interplay of genetic and environmental cofactors, it is difficult to discern the direct role STAT3 plays in the development of those conditions. Here, we report a mouse model of the *STAT3^R152W^* variant and describe its hematopoietic populations throughout adulthood. We observed profound changes in both innate and adaptive immunity, including increased splenic Th17 component consistent with a gain-of-function mutation, as described in the literature. At the same time, the mice did not develop obvious symptoms of autoimmunity. R152W mutants show lowered hemoglobin and hematocrit, indicating susceptibility to anemia, but also an increased number of thrombocytes, contradictory to reports of autoimmune thrombocytopenia. We showcase how those changes develop and wane in time, and the differences between male and female animals. Our findings paint the *STAT3^R152W^* variant as a cause of severe immune dysregulation, but only as a cofactor in the development of autoimmunity.

## 1. Introduction

STAT3 (Signal Transducer and Activator of Transcription 3) transcription factor has been closely linked to major organ function and dysfunction for years [1,2,3]. It is responsible for proper immune system activation and development, including immune cell differentiation and direct infection response [4,5,6,7]. Unsurprisingly, mutations in the *STAT3* gene are frequently present in populations suffering from cancer and autoimmunity, disorders where immunity is either impaired or overcharged [8,9]. Two terms were coined for the mutations modifying STAT3 activity—gain-of-function and loss-of-function (GOF and LOF)—attempting to classify the effects observed in the clinic [10]. However, this division presents us with a difficult task of categorizing the impact of a single mutation on the whole body while trying to account for dozens of confounding factors, such as other genetic variants and comorbidities. As such, this classification has recently been put into question [11]. Nonetheless, the *STAT3^R152W^* variant is widely considered to be a gain-of-function mutation. It is one of the most commonly found variants in patients with STAT3 GOF syndrome; for instance, a study identified it in 58 out of 191 patients recruited from 33 different countries [12,13]. It is a germline mutation substituting tryptophan for arginine in position 152. This mutation affects the coiled-coil domain of STAT3, which is responsible for receptor binding and predicted by modeling to have a stabilizing effect on the protein structure [14,15]. STAT3 activation by the R152W variant is supported by in vitro experiments showing enhanced *SOCS3* gene expression in mutant cells after cytokine stimulation [12]. Reported patients present with a variety of autoimmune disorders, such as type 1 diabetes, autoimmune thrombocytopenia and neutropenia, hemolytic anemia, as well as symptoms like delayed growth, caused by disrupted growth hormone signaling [13]. However, it is uncertain if the presence of the *STAT3^R152W^* variant alone can account for the development of a strong immune phenotype. To answer that question, for the first time, we introduced the *STAT3^R152W^* mutation into the endogenous *Stat3* gene in mice and performed a series of flow cytometry and CBC analyses over an extended period to confirm if the mutation induces aberrant immune system activation. Our research shows how various peripheral blood cell components change over a span of seven months, identifying which of those changes are compounded or attenuated by STAT3 activity and highlighting biological sex as an important factor in this and similar comparative studies.

## 2. Results

### 2.1. STAT3^R152W^ Mutants Present with Splenomegaly and Increased Splenic Th17 Component

**Figure 1:**
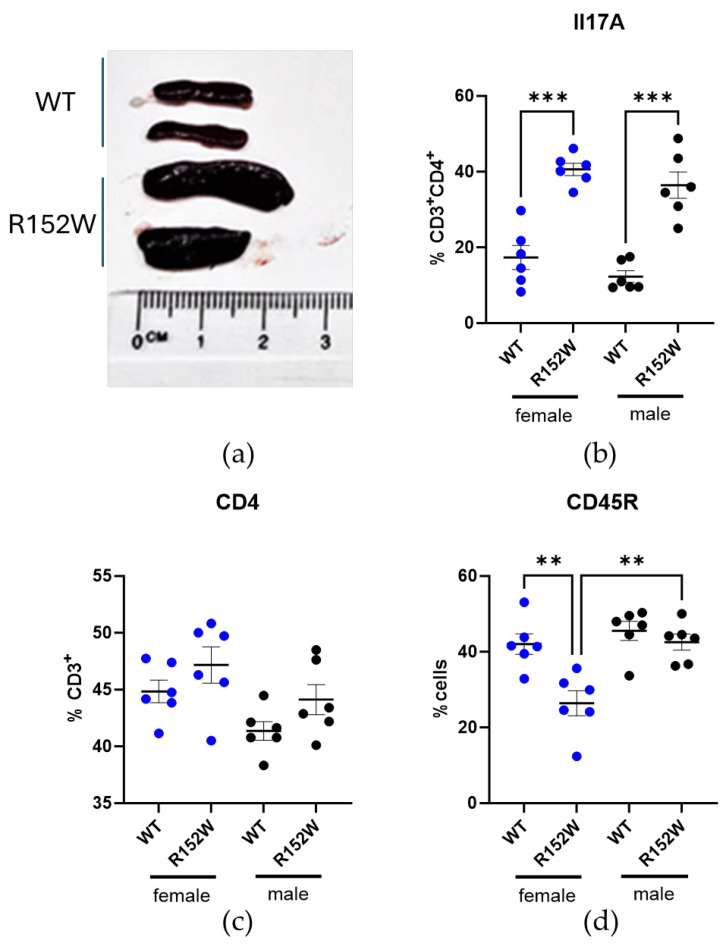
*STAT3^R152W^* mutants present with splenomegaly and increased splenic Th17 component. (**a**) Representative photograph of spleen size of R152W strain (bottom) and WT controls (top). (**b**–**d**) Population percentages of splenic CD4^+^CD8^+^Il17A^+^, CD3^+^CD4^+^, and CD45R^+^ cells in 6-month-old mice. *n* = 6, two-way ANOVA, ** *p* < 0.01, *** *p* < 0.001.

We first established whether the mutant strain displayed any STAT3 gain-of-function traits. Homozygous mice carrying the R152W variant (further called R152W) did not have a clear external phenotype except for a smaller body size, suggesting impaired growth hormone signaling that persisted into adulthood and was more significant in males (Figure S1b). They were also fertile, though with unsuccessful delivery being more common in this type than in wild-type C57Bl/6 mice. Upon autopsy, we discovered significantly enlarged spleens, reflecting splenomegaly reported in patients and common in autoimmune disorders like lupus or rheumatoid arthritis (Figure 1a). The qRT-PCR analysis revealed no significant upregulation in mRNA of *Stat3* or one of its downstream targets, *Socs3* (Figure S1c,d), in bulk tissue, suggesting cell-type-specific effects. In both male and female mice, we observed increased populations of CD3^+^CD4^+^Il17A^+^ cells, aligning with the enhanced potential for autoimmunity development, as Il-17A is a key mediator in associated processes (Figure 1b). While the difference in the percentage of splenic T cells remained below the statistical threshold, B cells (CD45R^+^) were depleted in female mutants compared to both WT females and R152W males (Figure 1c,d).

### 2.2. STAT3^R152W^ Variant Causes Long-Term Immune Shifts in Adaptive Immunity

**Figure 2:**
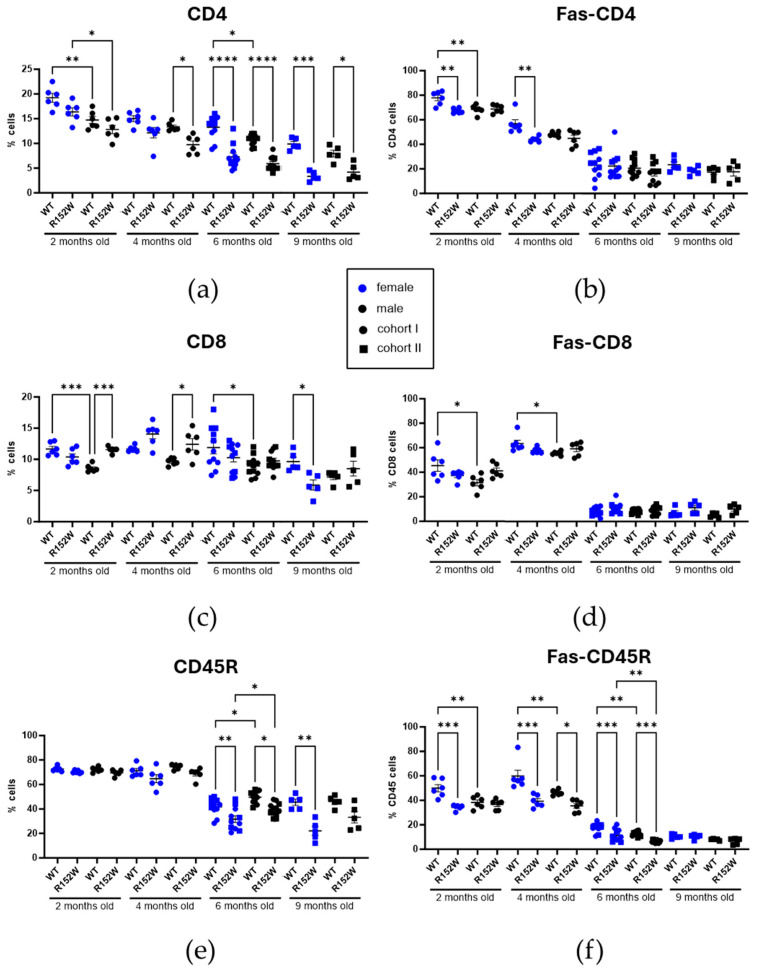
*STAT3^R152W^* variant causes long-term immune shifts in adaptive immunity (**a**–**f**). Cell populations in peripheral blood, as measured by flow cytometry: CD4^+^, CD8^+^, CD45R^+^, and their Fas-positive subsets. *n* = 6 (2–4 months), 11 (6 months), and 5 (9 months), two-way ANOVA, * *p* < 0.05, ** *p* < 0.01, *** *p* < 0.001, **** *p* < 0.00001.

We investigated two cohorts of age-matched R152W mice using complete blood count (CBC) and peripheral blood flow cytometry. The first cohort was analyzed at 2, 4, and 6 months of age (*n* = 6 per group, separately for males and females), and the second cohort was analyzed at 6 and 9 months (*n* = 5). Our analysis showed that CD4^+^ T-cell number decreased with age, while CD8^+^ population remained relatively stable; CD45R^+^ B-cell number decreased between 4 and 6 months of age (Figure 2 and Figure S2). CD4^+^ T-cell number was significantly sexually dimorphic at 2 months of age, being more abundant in females, and was trending this way until the 9-month timepoint. Across the timeline, the differences between WT and R152W in CD4^+^ and CD45R^+^ populations became greater, with the cell number decreasing in the mutants. Additionally, the Fas-CD4 population was only decreased at 2–4 months in R152W females, while the Fas-DC8 changes remained below the statistical threshold, and Fas-CD45R was lower in mutant mice until 6 months of age.

### 2.3. STAT3^R152W^ Mutant Mice Display Increased Presence of Innate Immunity Components

**Figure 3:**
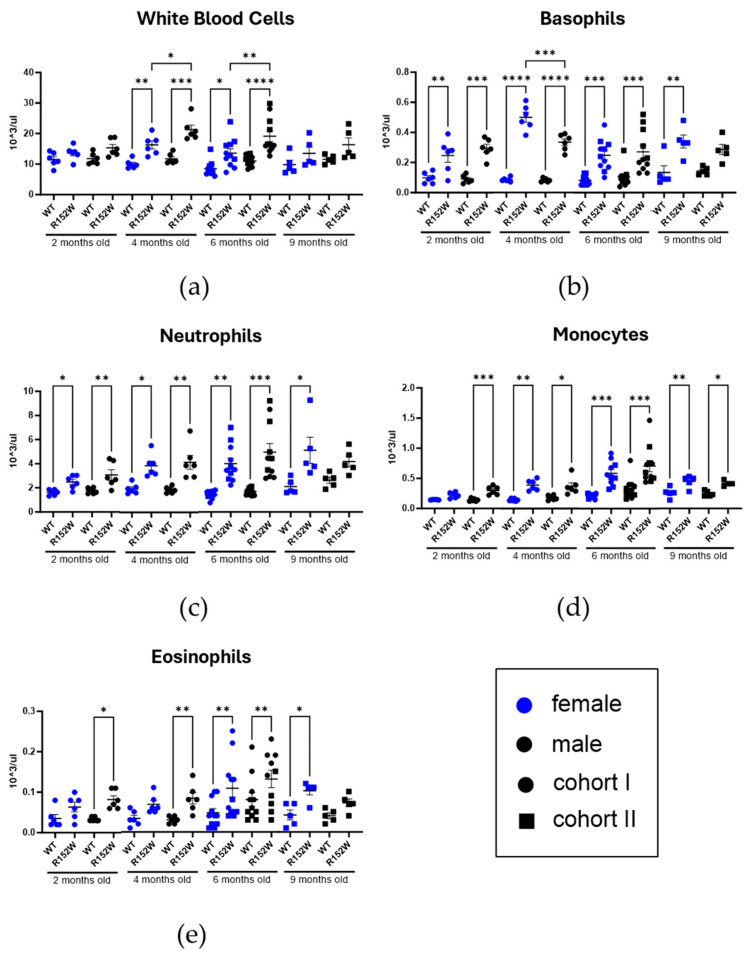
STAT3^R152W^ mutant mice display increased presence of innate immunity components (**a**–**e**). Cell populations in peripheral blood, as measured by CBC. *n* = 6 (2–4 months), 11 (6 months), and 5 (9 months), two-way ANOVA, * *p* < 0.05, ** *p* < 0.01, *** *p* < 0.001, **** *p* < 0.00001.

Next, we looked into innate immune populations using CBC (Figure 3). While autoimmunity is understandably associated with erroneous recognition of self-antigens, innate immunity plays an important role in the development and continuation of incorrect immune system reactivity. In addition to total white blood cells, populations of neutrophils, basophils, eosinophils, and monocytes were increased in the R152W strain compared to the control. We observed peak numbers at 6–9 months in both mutants and controls. Lastly, the statistically significant differences between strains emerged and waned faster in males.

### 2.4. STAT3^R152W^ Mutation Affects Thrombocyte and Erythrocyte Populations

**Figure 4:**
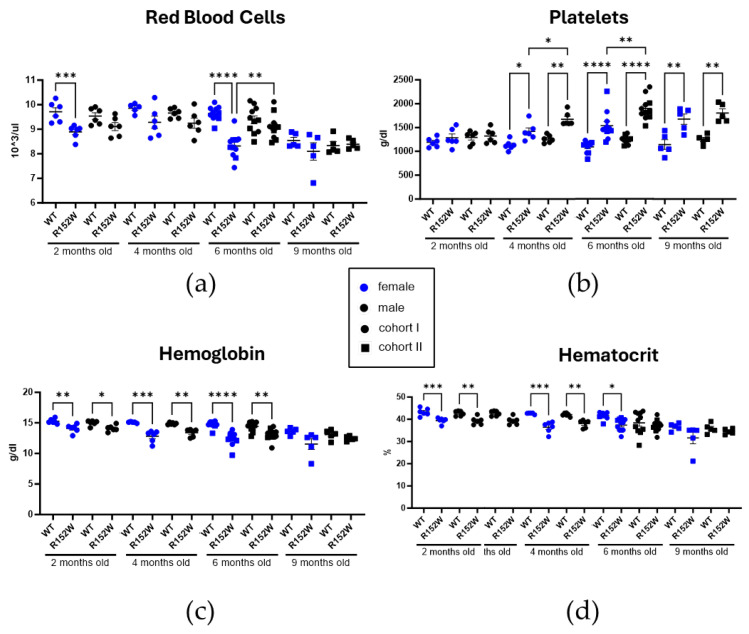
STAT3^R152W^ mutation affects thrombocyte and erythrocyte populations (**a**–**d**). Red blood cell and thrombocyte count, as well as hematocrit and hemoglobin values, as measured by CBC. *n* = 6 (2–4 months), 11 (6 months), and 5 (9 months), two-way ANOVA, * *p* < 0.05, ** *p* < 0.01, *** *p* < 0.001, **** *p* < 0.00001.

Lastly, using CBC, we assessed several red blood cell (RBC) and thrombocyte traits (Figure 4). While the RBC number in R152W mutants was lower, though inconsistently, than in controls, platelet number increased starting with the 4-month timepoint, and the association was stronger with males (Figure 4). Both hemoglobin and hematocrit were decreased in the mutants, more prominently in females than in males, and with the numbers becoming more similar between groups at the 9-month timepoint.

## 3. Discussion

In this manuscript, we showed the isolated effects of the *STAT3*^R152W^ mutation on the murine immune phenotype. While the literature identifies the R152W variant as a gain-of-function mutation, which is corroborated by in vitro experiments, it is likely too simplistic to attribute clinical presentations of patients harboring the mutation exclusively to STAT3 activity [12]. While the patient data is sparse due to confidentiality and the presentation is variable, our study has the advantage of showcasing a homogeneous population with minimal confounders. Additionally, diagnosing STAT3 disorders, which are more common in children than adults due to severity, is often delayed because of non-specific symptoms and limited availability of genetic testing [16]. As such, detailed and standardized immune population records at defined timepoints are not available.

Our results confirm that the *STAT3*^R152W^ variant can be classified as a gain-of-function. Mutant mice present with splenomegaly, and among splenic populations, Il-17A-positive T cells are more abundantly present in R152W mice. Il-17A is a known factor in the development of autoimmunity, and anti-Il-17A antibodies have been successfully used to treat autoimmune diseases like psoriasis [17,18,19]. We also know that the *Il17a* gene is under direct STAT3 transcriptional control, as the presence of its binding motif at the *Il17a* locus and direct promoter binding was demonstrated by chromatin immunoprecipitation [20,21].

However, despite that observation and homozygosity of the mutation, the mutant mice failed to develop a clearly manifesting disease phenotype. The overall decline in the number of peripheral T- and B-cell populations over time was expected and similar between experimental groups; however, at almost every step, it was affected by changes introduced by the R152W variant [22,23]. It was surprising to see that a hypothetical STAT3 gain-of-function system did not result in an increased overall CD4^+^ population, as their survival and pro-inflammatory effect are clearly linked to STAT3 activity [24,25,26]. Less abundant Fas-positive cell subsets might suggest a smaller probability of autoregulation and engagement in apoptotic pathways, but their impact on autoimmunity is not well-understood.

Further, we observed a unanimous increase in innate immunity compartments. Neutrophils, eosinophils, basophils, and monocytes have all established roles in autoimmunity, but the effects of their activity are very variable, perhaps reflecting *STAT3*^R152W^ patient phenotypes. The conditions range from eosinophilic vasculitis and basophil-driven angioedema to autoimmune liver disease and its key monocyte subsets, as well as multiple diseases affected by neutrophil extracellular traps [27,28,29,30]. With all those populations increased in R152W mice, this reinforces the hypothesis that STAT3 gain-of-function mutations might not be enough on their own to develop into a distinct condition with a defined diagnosis.

Finally, we presented thrombocyte and RBC numbers, as well as hematocrit and hemoglobin scores. Based on the literature, we expected thrombocytopenia, not thrombophilia, in the mutant mice. While increased platelets can be found in antiphospholipid antibody syndrome, a disease impacting pregnancy, one of the effects of the autoreactive antibodies is the downregulation of STAT3 in target tissues, making this association tenuous [31,32]. While the RBC-associated measures suggested increased potential for anemia development, as we would expect in a STAT3 gain-of-function mutant, the differences wane after the 6-month timepoint, again indicating a lack of chronic disease. While female patients are more likely to develop autoimmune hemolytic anemia, frequency should increase with age as well [33].

Our study has several limitations. While we wanted to show the baseline R152W mutant phenotype in this report, the changes in immune populations might not reflect STAT3 GOF syndrome patients, because the animals were housed in a sterile environment and their immune system was not challenged. While we indicate that our model represents STAT3 GOF, we do it indirectly, through observing changes in body size, splenomegaly, and expansion of the splenic IL-17A component. It is likely that a distinct immune cell subset and cytokine stimulation are required to quantifiably confirm changes in STAT3 dimerization and phosphorylation. Similarly, the mRNA expression of direct and indirect STAT3 targets needs to be studied individually in each altered immune component. Lastly, we were not able to conclusively state that the R152W variant positively or negatively affects cell survival and proliferation, as this differed between cell populations and was further confounded by sex and age.

In summary, we developed a mutant mouse strain harboring a *Stat3* variant that is common in the STAT3 GOF patient population and associated with autoimmune disease development. We demonstrated that the missense mutation is responsible for a large shift in immune cell populations, both splenic and peripheral, as well as a change in other blood parameters. While those imbalances might develop into disease upon the introduction of an additional factor like injury or infection, they had no effect on the basic functioning and lifespan of the mice. This is unlike the clinical setting, where *STAT3* mutations are found in patients displaying defined symptoms, often at a young age, and those symptoms are being linked directly to the *STAT3* variant itself. Our findings emphasize that not only are the immune changes caused by *STAT3*^R152W^ fluid in time, but the severity of their impact on phenotype might be dependent on yet unidentified cofactors.

## 4. Materials and Methods

### 4.1. Mice

All animals were housed and handled according to *the Guide for the Care and Use of Laboratory Animals* (8th edition), and all animal experiments were approved by the Animal Care and Use Committee (ACUC) of the National Institute of Diabetes and Digestive and Kidney Diseases (NIDDK, MD) and performed under the NIDDK animal protocol K089-LGP-23. CRISPR/Cas9-targeted mice were generated on a C57BL/6N background (Charles River, Wilmington, MA, USA) by the Transgenic Core of the National Heart, Lung, and Blood Institute (NHLBI). The *Stat3^R152W^* knock-in mouse line was generated using CRISPR/Cas9 technology. Briefly, a single guide RNA (sgRNA; CGATTACCTGCACTCGCTTC) was purchased from Synthego (Redwood City, CA, USA), and a single-strand oligonucleotide donor (CCAACAGCCGCCGTAGTGACAGAGAAGCAGCAGATGTTGGAGCAGCATCTTCAGGATGTC**T**GGAA*A*CG*C*GTGCAGGTAATCG GGCCTCACCCAGGGAGCCGCGCTGTGCTGTGCGGCTGCAGAGTGTGGTCCTGCGTGCACGC) was obtained from IDT (Coralville, IA, USA). In the donor sequence, the bold nucleotide (**T**) changed Codon 152 from CGG (encode Arg) to TGG (encode Trp). The italicized and underlined nucleotides (*A* and *C*) are silent mutations that do not cause amino acid changes but can help stop Cas9 from continuing to cut the target site after the donor is successfully knocked in. The sgRNA was first incubated with Cas9 protein (IDT) to form Cas9-sgRNA RNP complex, which was then co-electroporated together with the oligonucleotide donor into zygotes (549 total) collected from C57BL/6N mice using a Nepa21 electroporator (Nepa Gene Co., Ichikawa-City, Chiba, Japan) following procedures described by Kaneko [34]. The electroporated zygotes were cultured overnight in KSOM medium (Millipore Sigma, St. Louis, MO, USA, #MR-101-D) at 37 °C with 6% CO_2_. Those embryos that reached the two-cell stage of development (441 total) were implanted into the oviducts of pseudopregnant surrogate mothers (Swiss Webster mice from Charles River). Offspring were genotyped via PCR amplification followed by Sanger sequencing. Founder mice with the desired amino acid change were bred with wild-type C57BL/6 mice to expand the colony and for subsequent studies. After identifying the introduced mutation, heterozygous founders were bred with WT C57BL/6N mice. Representative genotyping results are presented in Figure S1. Homozygous mice from F2 and F3 generations and founder-derived age-matched WT controls were used in the study. A total of 44 mice were used to obtain the data described, and no exclusion criteria except age- and sex-matching were used. Animal age is stated within figures (cohorts of 2-, 4-, 6-, and 9-month-old mice). Euthanasia was performed by CO_2_ inhalation followed by cervical dislocation.

### 4.2. Complete Blood Count and Flow Cytometry

Blood was collected from the retro-orbital sinus into Eppendorf tubes in the presence of 5 mM ethylenediaminetetraacetic acid (EDTA, Sigma-Aldrich, St. Louis, MO, USA). Complete blood counts (CBC) were performed using an Element HT5 analyzer (Heska Corporation, Loveland, CO, USA). To perform surface antigen staining, remaining blood was washed using ACK buffer and the following antibodies were used to detect surface markers: PE Annexin V (BioLegend, San Diego, CA, USA, 640947), Brilliant Violet 421™ anti-mouse F4/80 (BioLegend 123132), Pacific Blue™ anti-mouse CD4 Antibody (BioLegend 100428), FITC anti-mouse CD62L Antibody (BioLegend 104406), PE/Cyanine5 anti-mouse/human CD45R/B220 Antibody (BioLegend 103210), PerCP/Cyanine5.5 anti-mouse/human CD11b Antibody (BioLegend 101228), PE/Cyanine7 anti-mouse CD8a Antibody (BioLegend 100722), APC anti-mouse/human CD44 Antibody (BioLegend 103012), Alexa Fluor^®^ 647 anti-mouse CD95 (Fas) Antibody (BioLegend 152620), and APC/Cyanine7 anti-mouse Ly-6G Antibody (BioLegend 127624). 7AAD was used as the viability dye. Intracellular Il-17A staining was performed by adding 45 min of incubation in fixation/permeabilization buffer (Invitrogen/eBioscience, San Diego, CA, USA) before washing and adding the following antibodies: APC/Cyanine7 anti-mouse CD8a Antibody (BioLegend 100714), PE/Cyanine5 anti-mouse/human CD45R/B220 Antibody (BioLegend 103210), APC anti-mouse IL-17A Monoclonal Antibody (ThermoFisher, Waltham, MA, USA, 14-7177-81), PE/Cyanine7 anti-mouse CD4 Antibody (BioLegend 100422), and FITC anti-mouse CD3 Antibody (BioLegend 100204). Flow cytometry data were acquired using the Cytek Aurora system (Cytek Biosciences, Fremont, CA, USA) and analyzed using SpectroFlo 3.3.0 software. Representative gating strategies are presented as Figures S3 and S4. Additional results, including measures not statistically significant and values identified as outliers, are included in Figures S5–S9.

### 4.3. Quantitative Real-Time PCR

Bulk splenic mRNA was isolated using PureLink™ RNA Mini Kit (Invitrogen), and 500 ng was transcribed into cDNA using SuperScript™ III First-Strand Synthesis SuperMix (Invitrogen, Carlsbad, CA, USA). qRT-PCR reaction was prepared with SsoAdvanced Universal Probes Supermix (Bio-Rad, Hercules, CA, USA) and the following Taqman probes (ThermoFisher): Stat3 (Mm01219775_m1), Socs3 (Mm00545913_s1), and Gapdh (4351309). PCR conditions were as follows: initial denaturation for 3 min at 95 °C, 35 cycles of 10 s at 95 °C, and 30 s at 60 °C.

### 4.4. Statistical Analysis

All samples were randomly selected and were limited only by genotype availability. Blinding was applied to CBC and flow cytometry analyses. For the comparison of samples, data were presented as the standard error of the mean. Data were analyzed for outliers (ROUT method, Q = 1%) and normal distribution (Shapiro–Wilk), followed by a two-way ANOVA with Tukey correction for multiple comparisons within a cohort and timepoint, or a non-parametric test. The F-test was used to compare the variance between groups. GraphPad PRISM 10.0.1 software was used for analysis. Values of * *p* < 0.05, ** *p* < 0.01, *** *p* < 0.001, **** *p*< 0.0001 were considered statistically significant. Group sizes are described in the figure description.

## Data Availability

Data are available in a publicly accessible repository; all CBC and flow cytometry data outputs were deposited in a Zenodo dataset of the same name and authorship as the manuscript (https://doi.org/10.5281/zenodo.18036659).

## Supplementary Materials

The following supporting information can be downloaded at: https://www.mdpi.com/article/10.3390/ijms27031587/s1.

## Abbreviations

The following abbreviations are used in this manuscript:

ACK Lysis Buffer	Ammonium–Chloride–Potassium Lysis Buffer
CBC	Complete Blood Count
GOF	Gain-of-function
LOF	Loss-of-function
RBC	Red Blood Cells
STAT3	Signal Transducer and Activator of Transcription 3

## References

[1] Hilfiker-Kleiner D., Hilfiker A., Drexler H. (2005). Many good reasons to have STAT3 in the heart. Pharmacol. Ther..

[2] Zhao J., Qi Y.F., Yu Y.R. (2021). STAT3: A key regulator in liver fibrosis. Ann. Hepatol..

[3] Pace J., Paladugu P., Das B., He J.C., Mallipattu S.K. (2019). Targeting STAT3 signaling in kidney disease. Am. J. Physiol. Ren. Physiol..

[4] Mackie J., Ma C.S., Tangye S.G., Guerin A. (2023). The ups and downs of STAT3 function: Too much, too little and human immune dysregulation. Clin. Exp. Immunol..

[5] Hillmer E.J., Zhang H., Li H.S., Watowich S.S. (2016). STAT3 signaling in immunity. Cytokine Growth Factor Rev..

[6] Ma C.S., Wong N., Rao G., Nguyen A., Avery D.T., Payne K., Torpy J., O’Young P., Deenick E., Bustamante J. (2016). Unique and shared signaling pathways cooperate to regulate the differentiation of human CD4+ T cells into distinct effector subsets. J. Exp. Med..

[7] Zhao C., Bai Y., Wang W., Amonkar G.M., Mou H., Olejnik J., Hume A.J., Mühlberger E., Lukacs N.W., Fearns R. (2024). Activation of STAT3-mediated ciliated cell survival protects against severe infection by respiratory syncytial virus. J. Clin. Investig..

[8] Zou S., Tong Q., Liu B., Huang W., Tian Y., Fu X. (2020). Targeting STAT3 in Cancer Immunotherapy. Mol. Cancer.

[9] Haapaniemi E.M., Kaustio M., Rajala H.L., van Adrichem A.J., Kainulainen L., Glumoff V., Doffinger R., Kuusanmäki H., Heiskanen-Kosma T., Trotta L. (2015). Autoimmunity, hypogammaglobulinemia, lymphoproliferation, and mycobacterial disease in patients with activating mutations in STAT3. Blood.

[10] Chandrasekaran P., Zimmerman O., Paulson M., Sampaio E.P., Freeman A.F., Sowerwine K.J., Hurt D., Alcántara-Montiel J.C., Hsu A.P., Holland S.M. (2016). Distinct mutations at the same positions of STAT3 cause either loss or gain of function. J. Allergy Clin. Immunol..

[11] Lodi L., Faletti L.E., Maccari M.E., Consonni F., Groß M., Pagnini I., Ricci S., Heeg M., Simonini G., Azzari C. (2022). STAT3-confusion-of-function: Beyond the loss and gain dualism. J. Allergy Clin. Immunol..

[12] Milner J.D., Vogel T.P., Forbes L., Ma C.A., Stray-Pedersen A., Niemela J.E., Lyons J.J., Engelhardt K.R., Zhang Y., Topcagic N. (2015). Early-onset lymphoproliferation and autoimmunity caused by germline STAT3 gain-of-function mutations. Blood.

[13] Leiding J.W., Vogel T.P., Santarlas V.G.J., Mhaskar R., Smith M.R., Carisey A., Vargas-Hernández A., Silva-Carmona M., Heeg M., Rensing-Ehl A. (2023). Monogenic early-onset lymphoproliferation and autoimmunity: Natural history of STAT3 gain-of-function syndrome. J. Allergy Clin. Immunol..

[14] Zhang T., Kee W.H., Seow K.T., Fung W., Cao X. (2000). The coiled-coil domain of Stat3 is essential for its SH2 domain-mediated receptor binding and subsequent activation induced by epidermal growth factor and interleukin-6. Mol. Cell Biol..

[15] Mansouri M., El Haddoumi G., Bendani H., Boumajdi N., Hakmi M., Abbou H., Bouricha E.M., Elgharbaoui B., Kartti S., El Jaoudi R. (2023). In Silico Analyses of All STAT3 Missense Variants Leading to Explore Divergent AD-HIES Clinical Phenotypes. Evol. Bioinform..

[16] Olbrich P., Freeman A.F. (2018). STAT1 and STAT3 mutations: Important lessons for clinical immunologists. Expert. Rev. Clin. Immunol..

[17] McGinley A.M., Sutton C.E., Edwards S.C., Leane C.M., DeCourcey J., Teijeiro A., Hamilton J.A., Boon L., Djouder N., Mills K.H.G. (2020). Interleukin-17A Serves a Priming Role in Autoimmunity by Recruiting IL-1β-Producing Myeloid Cells that Promote Pathogenic T Cells. Immunity.

[18] Huangfu L., Li R., Huang Y., Wang S. (2023). The IL-17 family in diseases: From bench to bedside. Signal Transduct. Target. Ther..

[19] Berry S.P.D., Dossou C., Kashif A., Sharifinejad N., Azizi G., Hamedifar H., Sabzvari A., Zian Z. (2022). The role of IL-17 and anti-IL-17 agents in the immunopathogenesis and management of autoimmune and inflammatory diseases. Int. Immunopharmacol..

[20] Chen Z., Laurence A., Kanno Y., Pacher-Zavisin M., Zhu B.M., Tato C., Yoshimura A., Hennighausen L., O’Shea J.J. (2006). Selective regulatory function of Socs3 in the formation of IL-17-secreting T cells. Proc. Natl. Acad. Sci. USA.

[21] Yang X.P., Ghoreschi K., Steward-Tharp S.M., Rodriguez-Canales J., Zhu J., Grainger J.R., Hirahara K., Sun H.W., Wei L., Vahedi G. (2011). Opposing regulation of the locus encoding IL-17 through direct, reciprocal actions of STAT3 and STAT5. Nat. Immunol..

[22] Morbach H., Eichhorn E.M., Liese J.G., Girschick H.J. (2010). Reference values for B cell subpopulations from infancy to adulthood. Clin. Exp. Immunol..

[23] Li M., Yao D., Zeng X., Kasakovski D., Zhang Y., Chen S., Zha X., Li Y., Xu L. (2019). Age related human T cell subset evolution and senescence. Immun. Ageing.

[24] Oh H.M., Yu C.R., Golestaneh N., Amadi-Obi A., Lee Y.S., Eseonu A., Mahdi R.M., Egwuagu C.E. (2011). STAT3 protein promotes T-cell survival and inhibits interleukin-2 production through up-regulation of Class O Forkhead transcription factors. J. Biol. Chem..

[25] Priceman S.J., Kujawski M., Shen S., Cherryholmes G.A., Lee H., Zhang C., Kruper L., Mortimer J., Jove R., Riggs A.D. (2013). Regulation of adipose tissue T cell subsets by Stat3 is crucial for diet-induced obesity and insulin resistance. Proc. Natl. Acad. Sci. USA.

[26] Liu X., Lee Y.S., Yu C.R., Egwuagu C.E. (2008). Loss of STAT3 in CD4+ T cells prevents development of experimental autoimmune diseases. J. Immunol..

[27] Buguth B., Bernarde J., Aggio D., Pimprikar A., Watt M., Westerink L., Persson J., Lloyd A. (2025). Estimation of Health Utility Values for Eosinophilic Granulomatosis with Polyangiitis. PharmacoEconomics Open.

[28] Kolkhir P., Muñoz M., Asero R., Ferrer M., Kocatürk E., Metz M., Xiang Y.K., Maurer M. (2022). Autoimmune chronic spontaneous urticaria. J. Allergy Clin. Immunol..

[29] Dyba M., Berezenko V., Zabara D., Bezpala A., Donskoi B. (2024). Monocyte subpopulations in children with autoimmune liver disease. Pathol. Res. Pract..

[30] Wigerblad G., Kaplan M.J. (2023). Neutrophil extracellular traps in systemic autoimmune and autoinflammatory diseases. Nat. Rev. Immunol..

[31] Knight J.S., Branch D.W., Ortel T.L. (2023). Antiphospholipid syndrome: Advances in diagnosis, pathogenesis, and management. BMJ.

[32] Mulla M.J., Myrtolli K., Brosens J.J., Chamley L.W., Kwak-Kim J.Y., Paidas M.J., Abrahams V.M. (2010). Antiphospholipid antibodies limit trophoblast migration by reducing IL-6 production and STAT3 activity. Am. J. Reprod. Immunol..

[33] Krumme A., Godby R., Blacketer C., Knoll C., Jackson L., Choudhry Z., Shah S. (2025). Prevalence and demographics of autoimmune hemolytic anemia in the United States. Blood.

[34] Kaneko T. (2023). Genome Editing in Mouse and Rat by Electroporation. Methods Mol. Biol..

