# Supplementary material for: STAT3R152W Mutation Model Reveals Temporal Changes in Hematopoietic Populations
Source: Int J Mol Sci. 2026 Feb 5;27(3):1587. doi: 10.3390/ijms27031587 (PMC12898827; doi:10.3390/ijms27031587)
Supplement: Supplementary file 1 [file ijms-27-01587-s001.zip › ijms-4090113-supplementary.pdf]

*Supplementary Figures*

# $STAT3^{R152W}$ Mutation Model Reveals Temporal Changes in Hematopoietic Populations

**Jakub Jankowski [1,*], Jichun Chen [2], Sung-Gwon Lee [1], Chengyu Liu [3], Neal Young [2] and Lothar Hennighausen [1]**

[1] Section of Genetics and Physiology, Laboratory of Cell and Molecular Biology, National Institute of Diabetes and Digestive and Kidney Diseases, US National Institutes of Health, Bethesda, MD 20892, USA; sunggwonl22@gmail.com (S.-G.L.); lotharh@niddk.nih.gov (L.H.)
[2] Hematology Branch, National Heart, Lung, and Blood Institute, US National Institutes of Health, Bethesda, MD 20892, USA; chenji@nhlbi.nih.gov (J.C.); youngns@nhlbi.nih.gov (N.Y.)
[3] Transgenic Core, National Heart, Lung, and Blood Institute, US National Institutes of Health, Bethesda, MD 20892, USA; liuch@nhlbi.nih.gov
* Correspondence: jakub.jankowski@nih.gov

**Abstract**
Inconsistent presentation of *STAT3* variants in the clinical setting makes them challenging to use in diagnostics and prevention of unfavorable outcomes. Patients harboring $STAT3^{R152W}$ variant display a range of autoimmune disorders, including type 1 diabetes, hemolytic anemia and thrombocytopenia. Because of a complex interplay of genetic and environmental cofactors, it is difficult to discern how direct of a role STAT3 plays in development of those conditions. Here, we report a mouse model of the $STAT3^{R152W}$ variant and describe its hematopoietic populations throughout adulthood. We observed vast changes in both innate and adaptive immunity, including increased splenic Th17 component consistent with a gain-of-function mutation as described in the literature. At the same time, the mice did not develop obvious symptoms of autoimmunity. R152W mutants show lowered hemoglobin and hematocrit, indicating susceptibility to anemia, but also increased number of thrombocytes, contradictory to reports of autoimmune thrombocytopenia. We showcase how those changes develop and wane in time, and the differences between male and female animals. Our findings paint the $STAT3^{R152W}$ variant as a cause of severe immune dysregulation, but only as a cofactor in development of autoimmunity.

**Keywords:** STAT3, germline mutation, missense mutation, immunology

**Supplementary Figure Descriptions**

**Figure S1.** Genotype and phenotype of $STAT3^{R152W}$ mutants. Representative genotyping of the mutant strain, with the missense mutation marked in red, and silent substitutions allowing for identification of successful recombination in blue (a), body size at 6 months of age (b), *Stat3* and *Socs3* mRNA expression in the spleen relative to mean female WT expression (c,d). 2-way ANOVA, $n$ = 7 (female) and 9 (male), *** $p < 0.001$, bar = SEM

**Figure S2.** Figure 2. $STAT3^{R152W}$ variant causes long-term immune shifts in adaptive immunity (a-f) Cell numbers in peripheral blood as measured by flow cytometry: $CD4^+$, $CD8^+$, $CD45R^+$, and their Fas-positive subsets. $n$ = 6 (2-4 months), 11(6 months) and 5 (9 months), 2-way ANOVA, * $P < 0.05$, ** $P < 0.01$, *** $P < 0.001$ **** $P < 0.00001$

**Figure S3.** Representative gating strategy for flow cytometry – surface staining.

**Figure S4.** Representative gating strategy for flow cytometry – intracellular staining.

**Figure S5.** Full CBC and flow cytometry results for all measures, including datapoints excluded in manuscript, in cohort 1, at 2 months old (a-z). $n$ = 6, * $p < 0.05$, ** $p < 0.01$, *** $p < 0.001$, **** $p < 0.0001$, bar = SEM

**Figure S6.** Full CBC and flow cytometry results for all measures, including datapoints excluded in manuscript, in cohort 1, at 4 months old (a-z). $n$ = 6, * $p < 0.05$, ** $p < 0.01$, *** $p < 0.001$, **** $p < 0.0001$, bar = SEM

**Figure S7.** Full CBC and flow cytometry results for all measures, including datapoints excluded in manuscript, in cohort 1, at 6 months old (a-z). $n$ = 6, * $p < 0.05$, ** $p < 0.01$, *** $p < 0.001$, **** $p < 0.0001$, bar = SEM

**Figure S8.** Full CBC and flow cytometry results for all measures, including datapoints excluded in manuscript, in cohort 2, at 6 months old (a-z). $n$ = 5, * $p < 0.05$, ** $p < 0.01$, *** $p < 0.001$, bar = SEM

**Figure S9.** Full CBC and flow cytometry results for all measures, including datapoints excluded in manuscript, in cohort 2, at 9 months old (a-z). $n$ = 5, * $p < 0.05$, ** $p < 0.01$, *** $p < 0.001$, bar = SEM

Figure S1

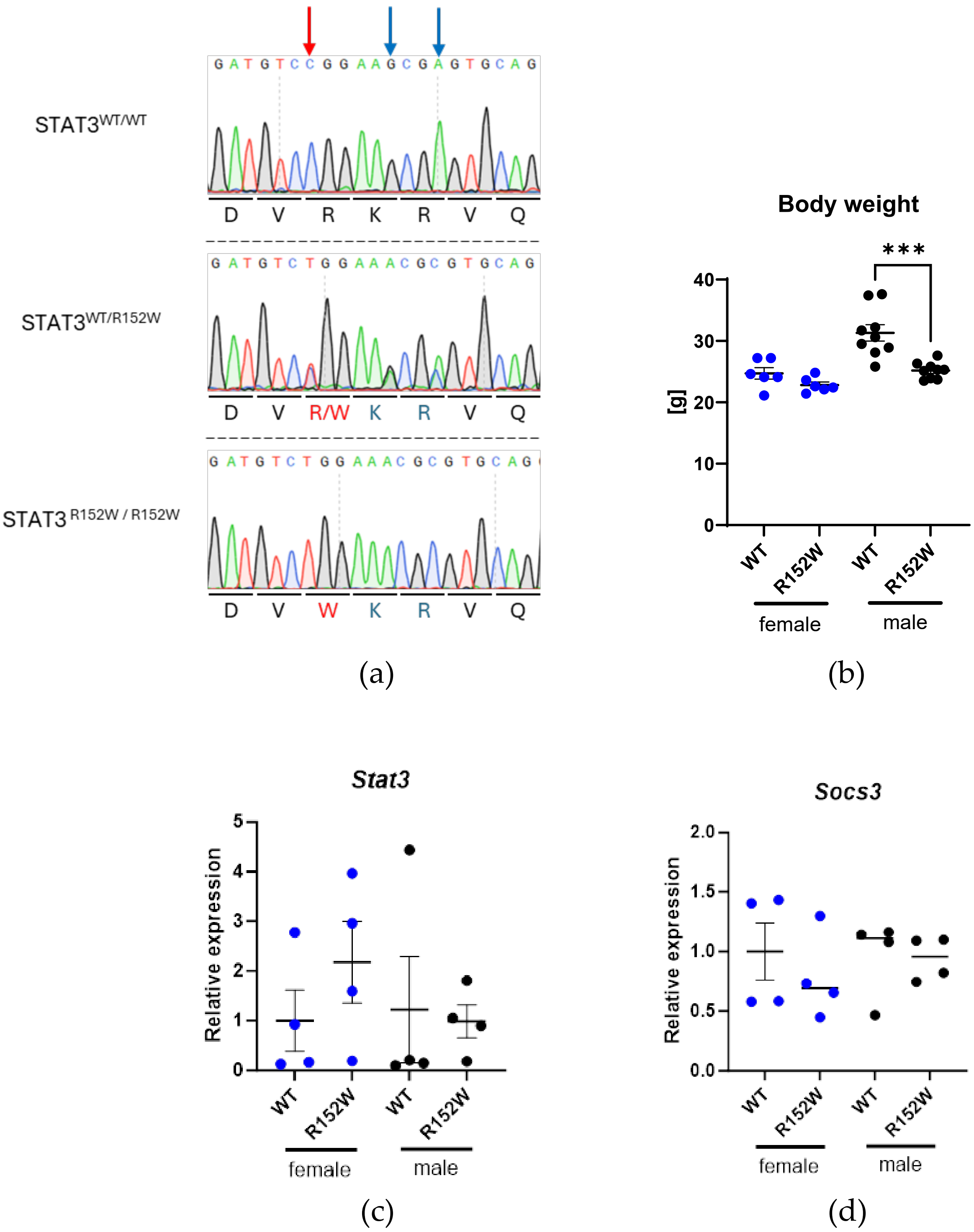
STAT3^WT/WT
G A T G T C C G G A A G C G A G T G C A G
D V R K R V Q
STAT3^WT/R152W
G A T G T C T G G A A A C G C G T G C A G
D V R/W K R V Q
STAT3 R152W / R152W
D V W K R V Q
(a)
Body weight
***
[g]
40
30
20
10
0
WT
R152W
WT
R152W
female
male
(b)
Stat3
Relative expression
5
4
3
2
1
0
WT
R152W
WT
R152W
female
male
(c)
Socs3
Relative expression
2.0
1.5
1.0
0.5
0.0
WT
R152W
WT
R152W
female
male
(d)

Figure S2

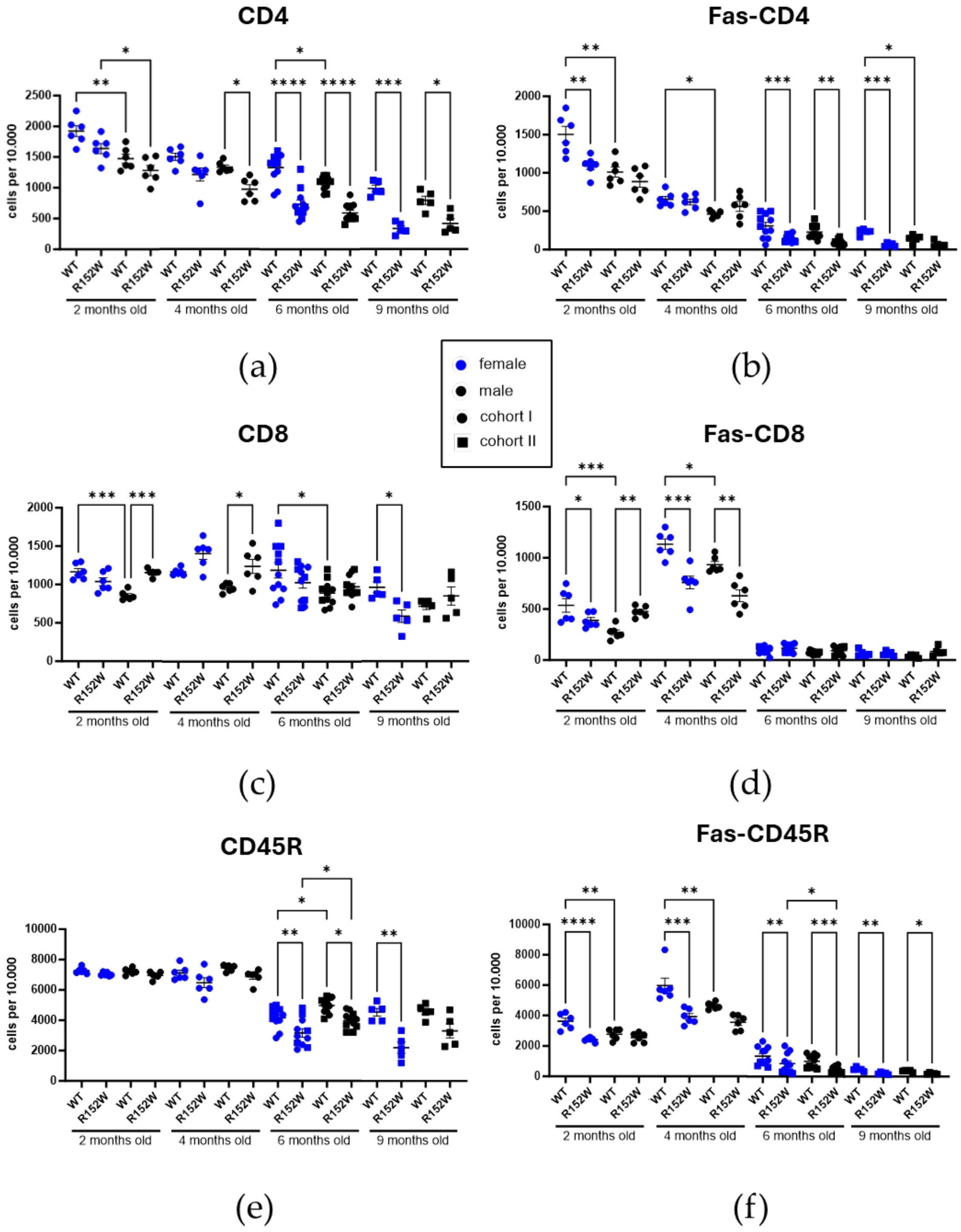
CD4
Fas-CD4
CD8
Fas-CD8
CD45R
Fas-CD45R
cells per 10.000
WT
R152W
2 months old
4 months old
6 months old
9 months old
female
male
cohort I
cohort II
(a)
(b)
(c)
(d)
(e)
(f)

Figure S3

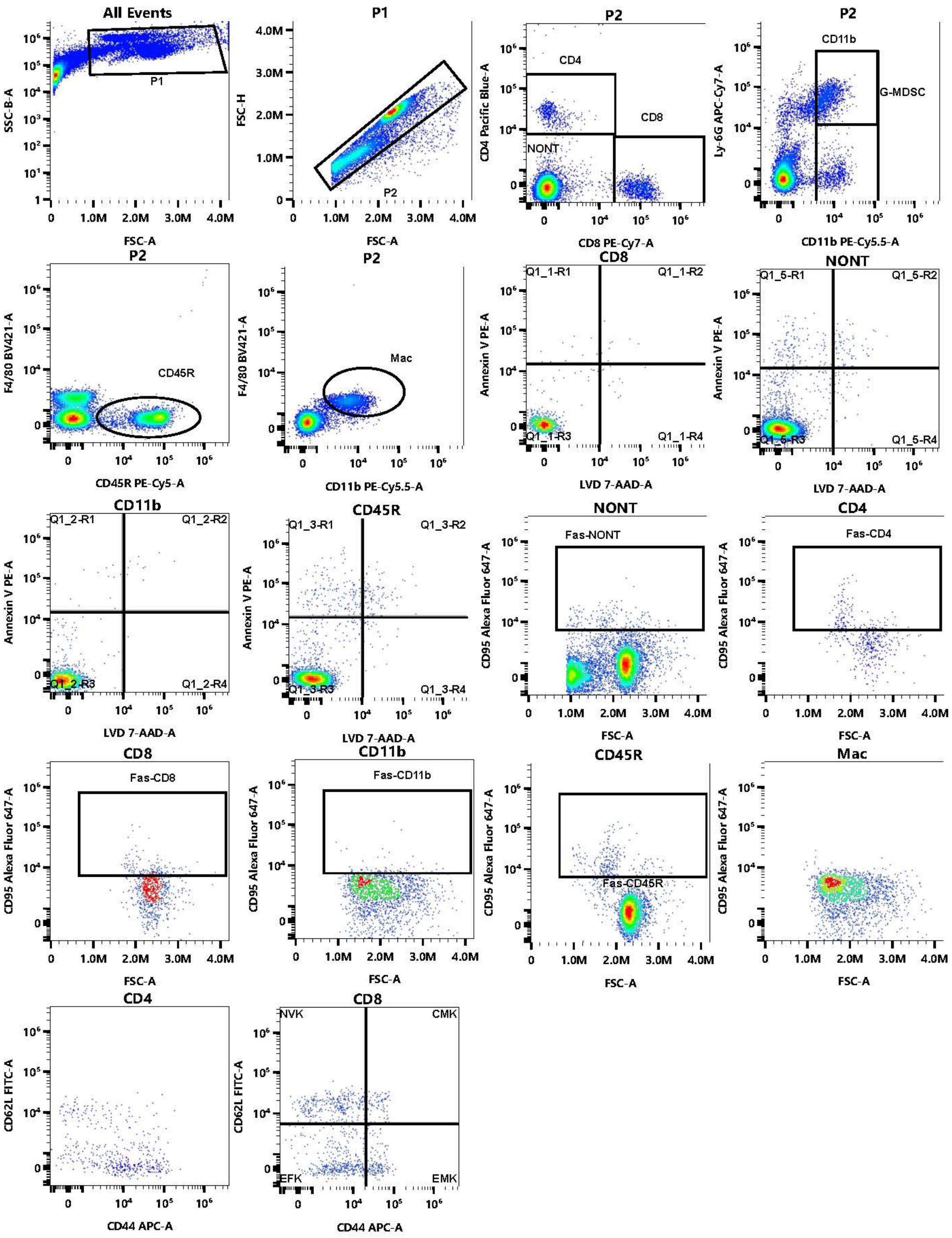
All Events
P1
P2
CD4
CD8
NONT
CD11b
G-MDSC
CD45R
Mac
Q1_1-R1
Q1_1-R2
Q1_1-R3
Q1_1-R4
Q1_5-R1
Q1_5-R2
Q1_5-R3
Q1_5-R4
Q1_2-R1
Q1_2-R2
Q1_2-R3
Q1_2-R4
Q1_3-R1
Q1_3-R2
Q1_3-R3
Q1_3-R4
Fas-NONT
Fas-CD4
Fas-CD8
Fas-CD11b
Fas-CD45R
NVK
CMK
EFK
EMK
SSC-B-A
FSC-A
FSC-H
CD4 Pacific Blue-A
CD8 PE-Cy7-A
Ly-6G APC-Cy7-A
CD11b PE-Cy5.5-A
F4/80 BV421-A
CD45R PE-Cy5-A
Annexin V PE-A
LVD 7-AAD-A
CD95 Alexa Fluor 647-A
CD62L FITC-A
CD44 APC-A

Figure S4

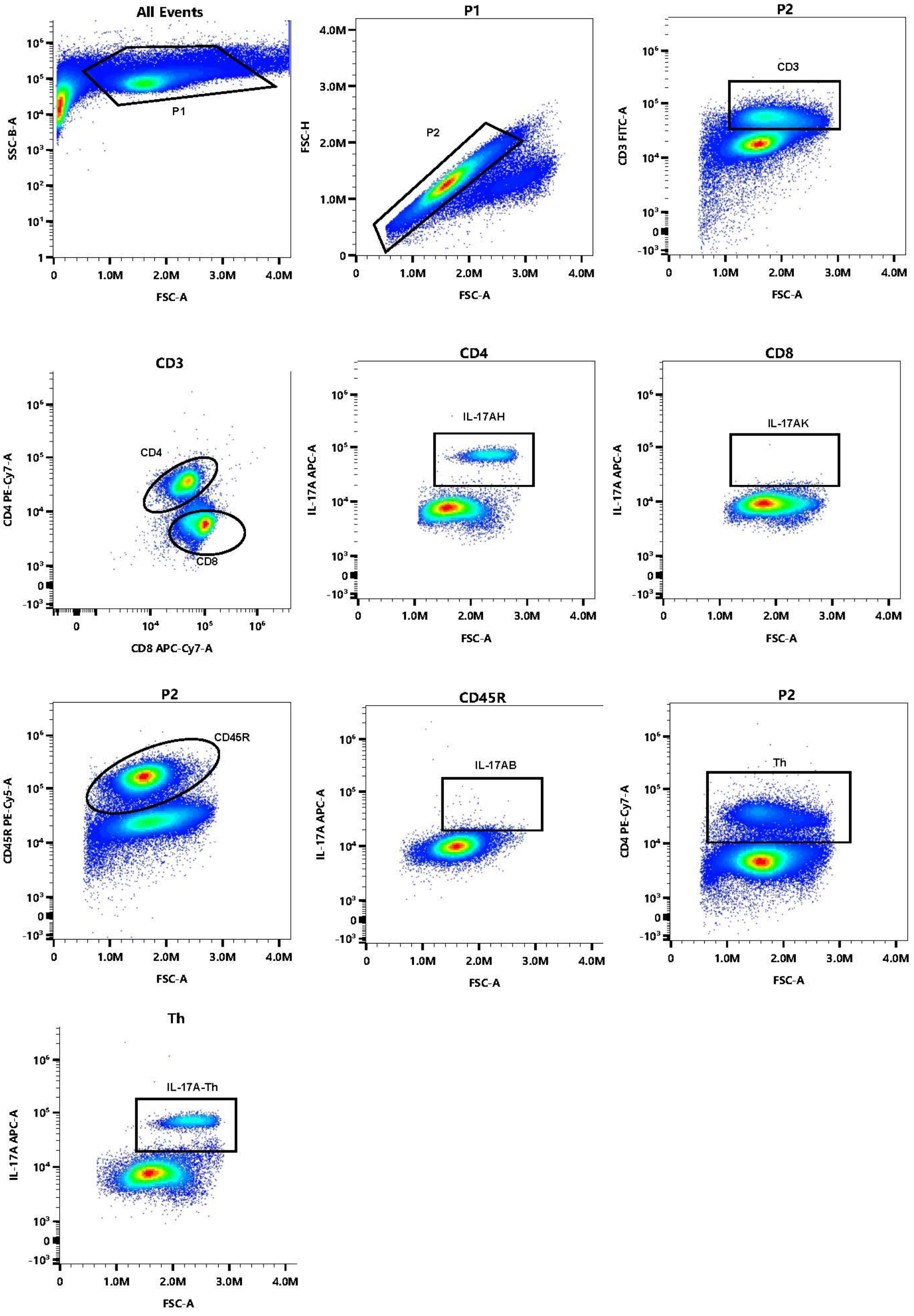
All Events
P1
SSC-B-A
FSC-A
P1
P2
FSC-H
FSC-A
P2
CD3
CD3 FITC-A
FSC-A
CD3
CD4
CD8
CD4 PE-Cy7-A
CD8 APC-Cy7-A
CD4
IL-17AH
IL-17A APC-A
FSC-A
CD8
IL-17AK
IL-17A APC-A
FSC-A
P2
CD45R
CD45R PE-Cy5-A
FSC-A
CD45R
IL-17AB
IL-17A APC-A
FSC-A
P2
Th
CD4 PE-Cy7-A
FSC-A
Th
IL-17A-Th
IL-17A APC-A
FSC-A

Figure S5

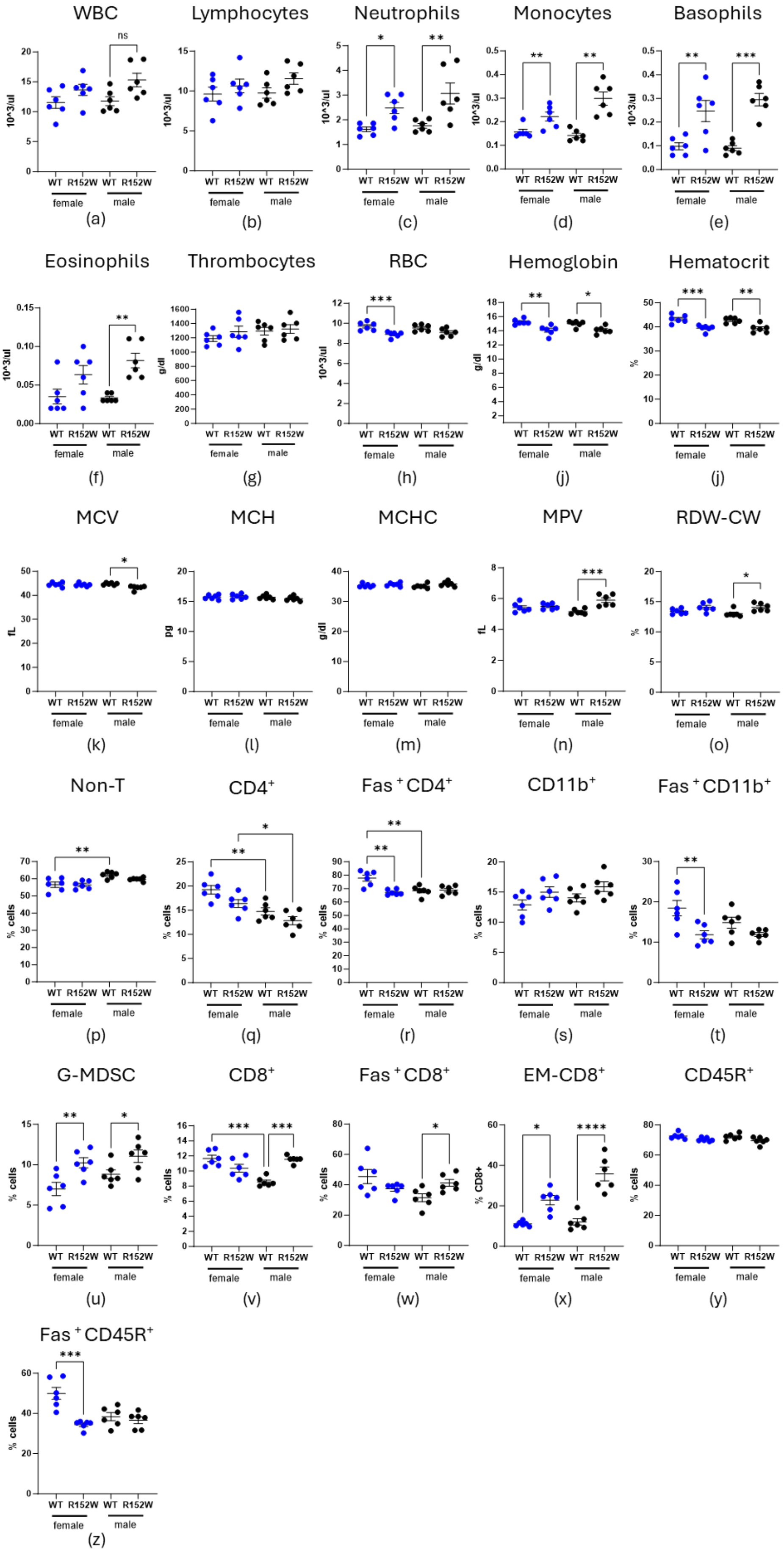
WBC
Lymphocytes
Neutrophils
Monocytes
Basophils
(a)
(b)
(c)
(d)
(e)
Eosinophils
Thrombocytes
RBC
Hemoglobin
Hematocrit
(f)
(g)
(h)
(j)
(j)
MCV
MCH
MCHC
MPV
RDW-CW
(k)
(l)
(m)
(n)
(o)
Non-T
CD4+
Fas+CD4+
CD11b+
Fas+CD11b+
(p)
(q)
(r)
(s)
(t)
G-MDSC
CD8+
Fas+CD8+
EM-CD8+
CD45R+
(u)
(v)
(w)
(x)
(y)
Fas+CD45R+
(z)
WT R152W WT R152W
female
male
10^3/ul
g/dl
fL
pg
%
% cells
% CD8+
ns

Figure S6

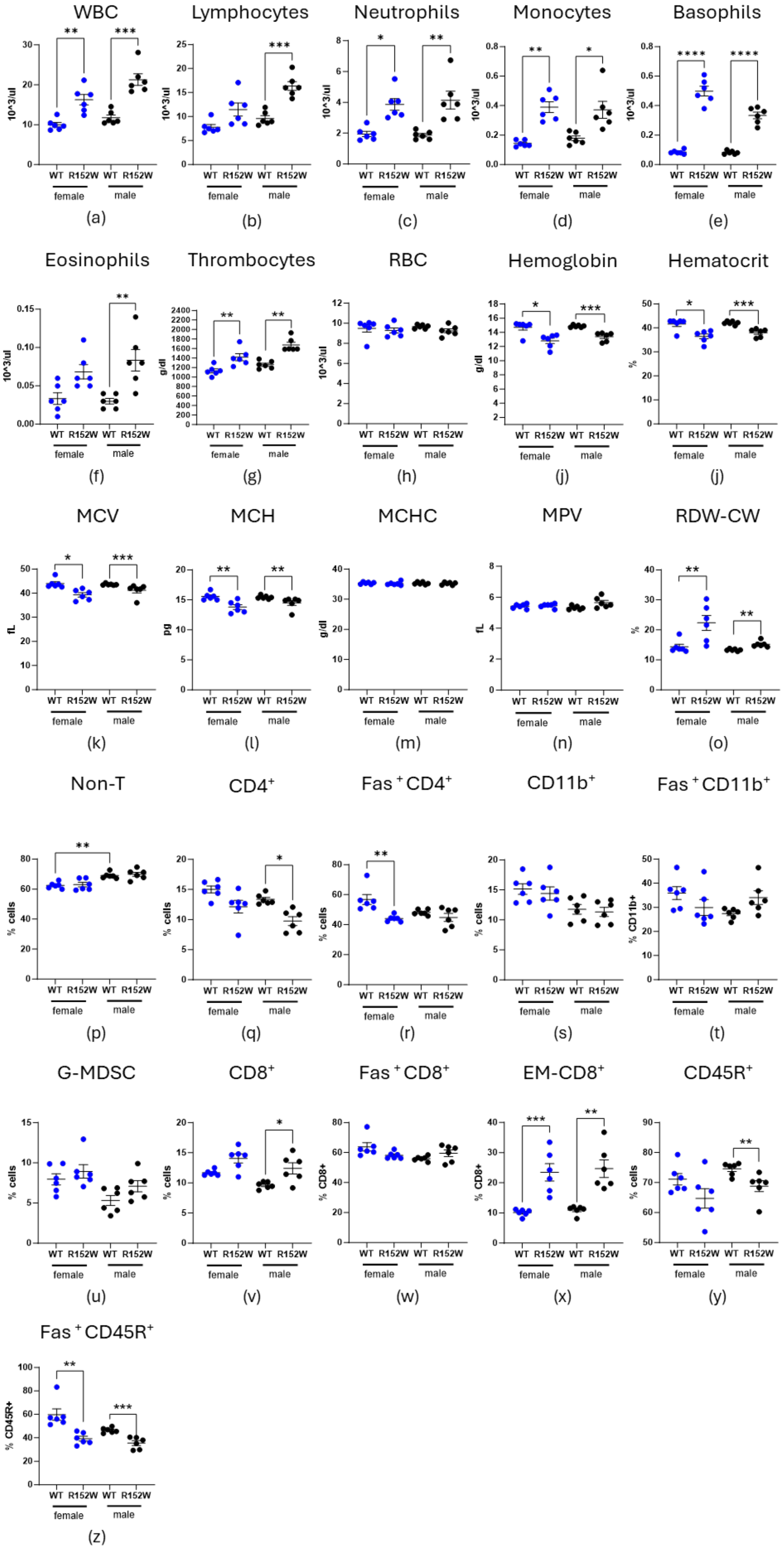
WBC
Lymphocytes
Neutrophils
Monocytes
Basophils
10^3/ul
WT R152W WT R152W
female male
(a)
(b)
(c)
(d)
(e)
Eosinophils
Thrombocytes
RBC
Hemoglobin
Hematocrit
10^3/ul
g/dl
%
(f)
(g)
(h)
(j)
(j)
MCV
MCH
MCHC
MPV
RDW-CW
fL
pg
g/dl
%
(k)
(l)
(m)
(n)
(o)
Non-T
CD4+
Fas+CD4+
CD11b+
Fas+CD11b+
% cells
% CD11b+
(p)
(q)
(r)
(s)
(t)
G-MDSC
CD8+
Fas+CD8+
EM-CD8+
CD45R+
% cells
% CD8+
(u)
(v)
(w)
(x)
(y)
Fas+CD45R+
% CD45R+
(z)

Figure S7

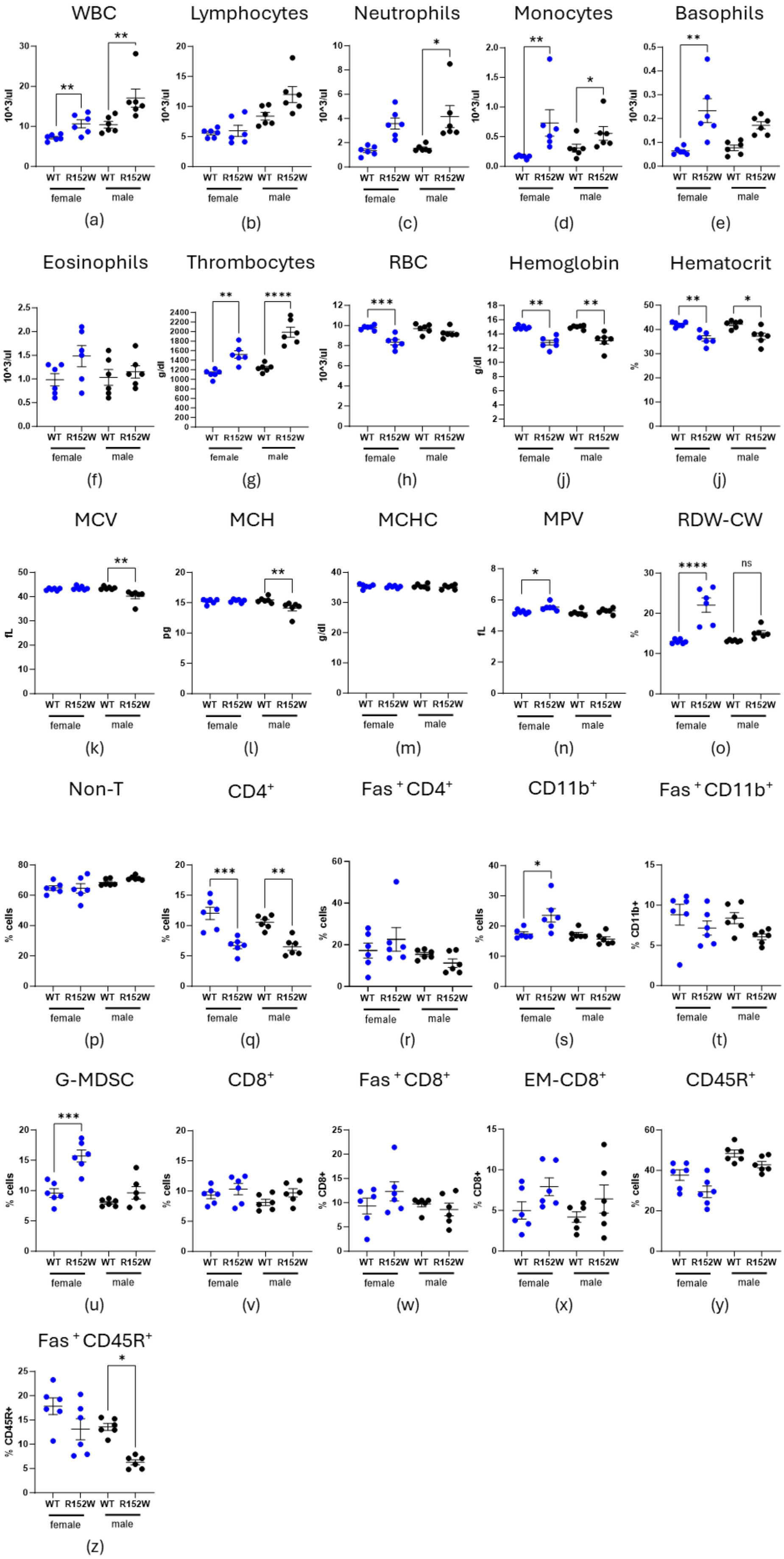

WBC
10^3/ul
(a)
Lymphocytes
10^3/ul
(b)
Neutrophils
10^3/ul
(c)
Monocytes
10^3/ul
(d)
Basophils
10^3/ul
(e)
Eosinophils
10^3/ul
(f)
Thrombocytes
g/dl
(g)
RBC
10^3/ul
(h)
Hemoglobin
g/dl
(j)
Hematocrit
%
(j)
MCV
fL
(k)
MCH
pg
(l)
MCHC
g/dl
(m)
MPV
fL
(n)
RDW-CW
%
ns
(o)
Non-T
% cells
(p)
CD4+
% cells
(q)
Fas+CD4+
% cells
(r)
CD11b+
% cells
(s)
Fas+CD11b+
% CD11b+
(t)
G-MDSC
% cells
(u)
CD8+
% cells
(v)
Fas+CD8+
% CD8+
(w)
EM-CD8+
% CD8+
(x)
CD45R+
% cells
(y)
Fas+CD45R+
% CD45R+
(z)
WT
R152W
female
male

Figure S8

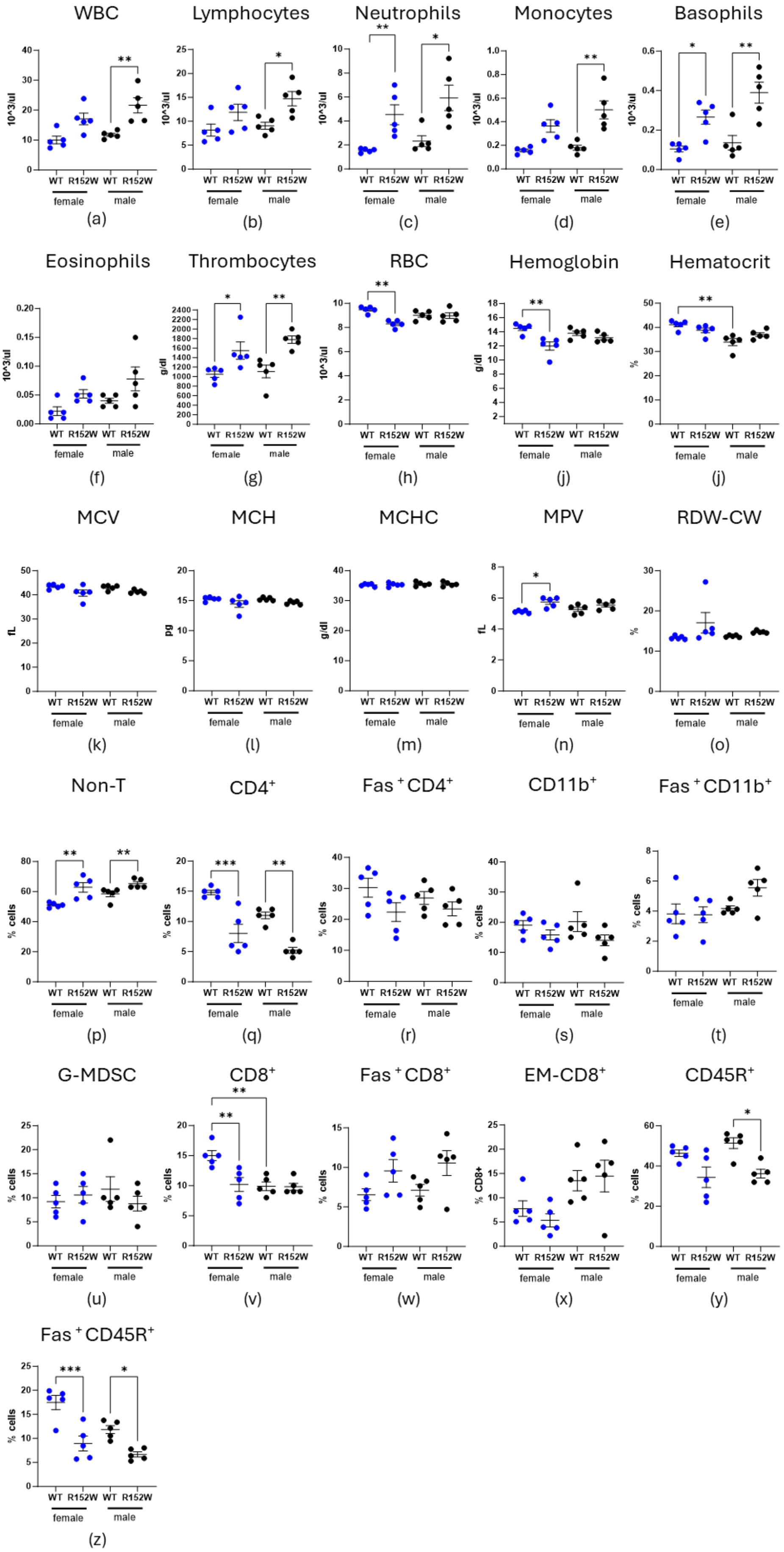

WBC
Lymphocytes
Neutrophils
Monocytes
Basophils
(a)
(b)
(c)
(d)
(e)
Eosinophils
Thrombocytes
RBC
Hemoglobin
Hematocrit
(f)
(g)
(h)
(j)
(j)
MCV
MCH
MCHC
MPV
RDW-CW
(k)
(l)
(m)
(n)
(o)
Non-T
CD4+
Fas+CD4+
CD11b+
Fas+CD11b+
(p)
(q)
(r)
(s)
(t)
G-MDSC
CD8+
Fas+CD8+
EM-CD8+
CD45R+
(u)
(v)
(w)
(x)
(y)
Fas+CD45R+
(z)
WT R152W WT R152W
female
male
10^3/ul
g/dl
%
fL
pg
% cells
% CD8+

Figure S9

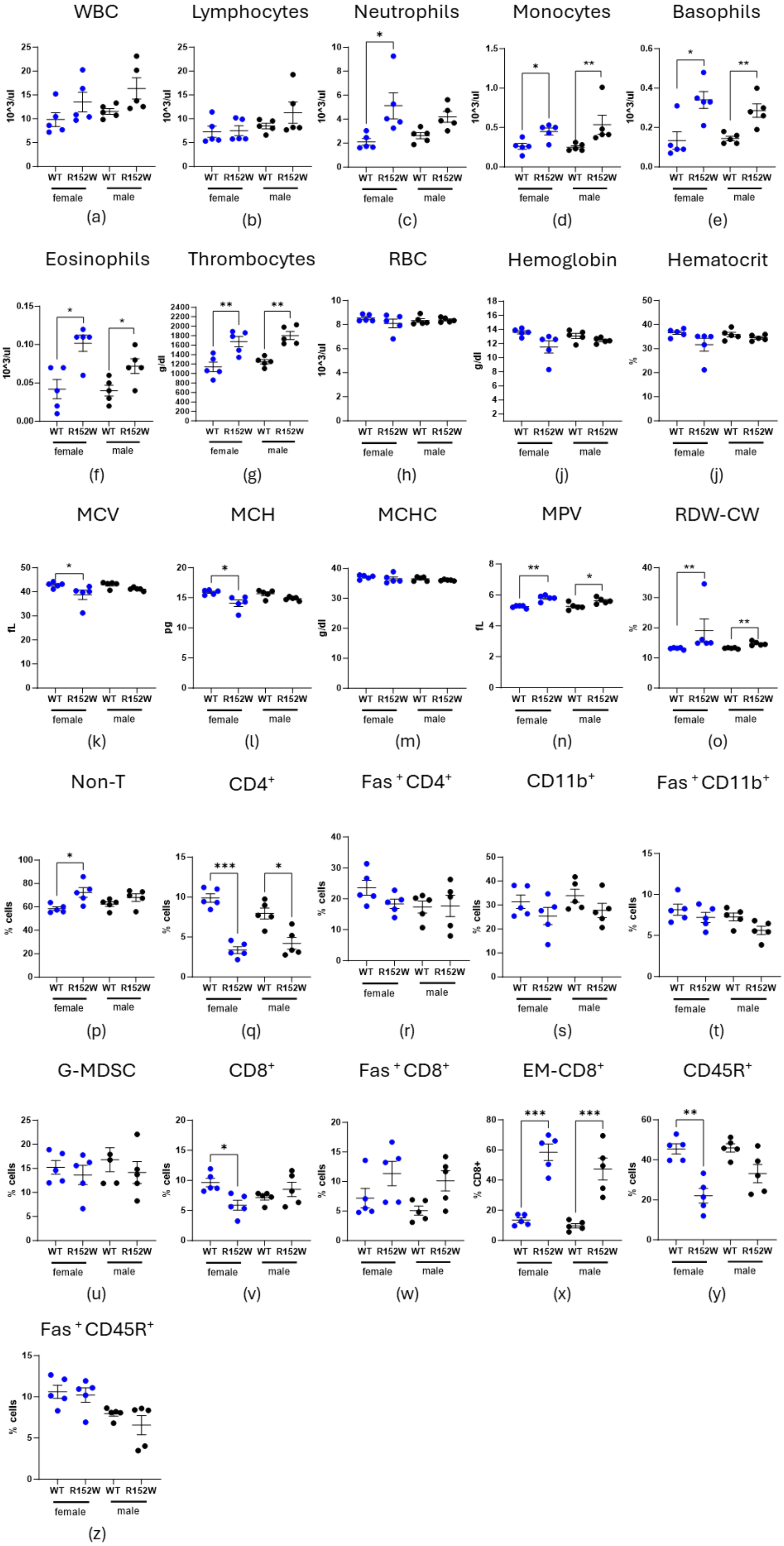
WBC
Lymphocytes
Neutrophils
Monocytes
Basophils
(a)
(b)
(c)
(d)
(e)
Eosinophils
Thrombocytes
RBC
Hemoglobin
Hematocrit
(f)
(g)
(h)
(j)
(j)
MCV
MCH
MCHC
MPV
RDW-CW
(k)
(l)
(m)
(n)
(o)
Non-T
CD4+
Fas+CD4+
CD11b+
Fas+CD11b+
(p)
(q)
(r)
(s)
(t)
G-MDSC
CD8+
Fas+CD8+
EM-CD8+
CD45R+
(u)
(v)
(w)
(x)
(y)
Fas+CD45R+
(z)
WT R152W WT R152W
female
male
10^3/ul
g/dl
%
fL
pg
% cells
% CD8+
